# Bovine serum albumin aggravates macrophage M1 activation and kidney injury in heterozygous Klotho-deficient mice via the gut microbiota-immune axis

**DOI:** 10.7150/ijbs.56424

**Published:** 2021-02-02

**Authors:** Lingyun Lai, Yi Li, Jianjun Liu, Lei Luo, Jianguo Tang, Jun Xue, Te Liu

**Affiliations:** 1 Division of Nephrology, Huashan Hospital, Fudan University, Shanghai 200040, China.; 2 Division of Nephrology, Shuguang Hospital, Shanghai University of Traditional Chinese Medicine, Shanghai 201203, China.; 3 Trauma-Emergency & Critical Care Medicine Center, Shanghai Fifth People's Hospital, Fudan University, Shanghai 200240, China.; 4 Shanghai Geriatric Institute of Chinese Medicine, Shanghai University of Traditional Chinese Medicine, Shanghai 200031, China.; 5 Department of Pathology, Yale University School of Medicine, New Haven, Connecticut 06520, USA.

**Keywords:** gut microbiota, macrophage M1, Klotho, Nrf2/NF-κB pathway, kidney injury

## Abstract

Klotho expression abnormalities induces kidney injury and chronic kidney disease, however, the underlying mechanism remains unclear. Here, Klotho^+/-^ mice and wild-type mice were treated with low-dose bovine serum albumin (BSA). Pathological examination demonstrated that the area of glomerular collagen deposition and fibrosis in BSA-Kl^-/+^ mice was significantly larger than that in BSA-WT mice. The serum levels of superoxide dismutase, malondialdehyde, creatinine, and urea in BSA-Kl^-/+^ mice were significantly increased. Sequencing of gut microbiota 16S rRNA v3-v4 region indicated that BSA-Kl^-/+^ mice showed a significantly higher relative abundance of the genera *Dubosiella*, *Akkermansia*, *Alloprevotella*, and *Lachnospiraceae* and a significantly lower relative abundance of the genera *Allobaculum* and *Muribaculaceae* than BSA-WT mice. KEGG analysis revealed that the metabolic pathways of signal transduction, xenobiotic biodegradation and metabolism, and lipid metabolism increased significantly in BSA-Kl^-/+^ mice. Flow cytometry showed that the proportion of CD68^+^/CD11b^+^ cells in the peripheral blood was significantly higher in BSA-KL^-/+^ mice than that in BSA-WT mice. qPCR and western blot suggested that Klotho and Nrf2 expression in MΦ1 cells of BSA-KL^-/+^ mice was significantly decreased. Thus, the findings suggest during the immune activation and chronic inflammation induced by the gut microbiota imbalance in Klotho-deficient mice treated to BSA, disrupted expression of proteins in the Nrf2/NF-κB signaling pathway in monocyte-derived macrophage M1 cells leads to the aggravation of inflammation and kidney injury.

## Introduction

Chronic kidney disease affects approximately 10% of the global population and has a financial impact of approximately $48 billion per year in the United States alone 1, 2. Hypertension is an important risk factor for CKD, and approximately 85-90% of patients with CKD stage 3-5 have hypertension 1, 2. Increasing evidence indicates an important role of the gut microbiota in the development of hypertension and CKD 1-3. The human gut microbiota comprises more than 100 trillion microbial cells, including aerobic and anaerobic species and gram-positive and gram-negative species 4. The gut microbiota constantly communicates with vital organ systems of the host, such as the brain, bone marrow, vasculature, kidney, immune system, and autonomic nervous system 1, 5, 6. Trimethylamine-N-oxide, short-chain fatty acids, and inflammatory factors derived from the gut microbes may induce changes in arteries, kidneys, and blood pressure 4. In addition, bacterial 16S rDNA amplification and DNA pyrosequencing suggest that the DNA of gut microbiota is present in the plasma of CKD patients on chronic hemodialysis 7, 8, and the levels of bacterial DNA is correlated with increased plasma inflammatory marker levels 7, 8. Our previous study revealed that ranitidine and finasteride inhibit the synthesis and release of trimethylamine N-oxide and mitigate its cardiovascular and kidney damage through modulating gut microbiota 9. Indoxyl sulfate is a typical uremic toxin that is not efficiently removed by hemodialysis; modulation of indoxyl sulfate production in the gut microbiota is a promising strategy for decreasing indoxyl sulfate concentration, thus delaying CKD progression 10. Therefore, exploring the relationship between gut microbiota and kidney disease important for the development of effective therapeutic drugs.

Klotho (*KL*) was initially identified as an anti-aging gene, mainly expressed in the kidney and cerebral choroid plexus 11-16. Human *KL* gene encodes α-Klotho, a multifunctional protein that regulates the metabolism of phosphate, calcium, and vitamin D and acts as a hormone 14, 17-22. A human *KL* gene point mutation is associated with hypertension and kidney disease, suggesting that KL maintains normal kidney function. *Kl* overexpression in mice prolongs their life 11, 13, 19, 23. *KL* gene knockout accelerates aging and shortens the life span 18-22. The *KL* gene encodes three protein forms: full-length transmembrane KL, truncated soluble KL, and secretory KL (sKL). Soluble KL is produced by releasing the extracellular domain of transmembrane KL, whereas sKL is produced by selective RNA splicing 12-14, 24. sKL secreted from the cells is found in blood, urine, and cerebrospinal fluid 12-14; it is an endocrine factor that targets distal organs and regulates the activity of cell surface ion channels and transporters 12-14. Klotho deficiency is closely associated with kidney disease. In patients with CKD stages 1-5, the combination of higher C-terminal fibroblast growth factor 23 and lower sKL levels were associated with adverse clinical outcomes 25. High plasma phosphate and Klotho deficiency contribute to kidney epithelial senescence and kidney fibrosis, an important pathological feature in the aging kidney and chronic kidney disease 26. In *Kl*^-/-^ mice, severe skeletal muscle atrophy was observed, and autophagy-lysosome pathway was activated. The signal transduction activity of mTOR was inhibited, likely due to the lack of essential amino acids in KL-deficient glomeruli 18. Chen and others reported that RGFP966, a selective inhibitor of histone deacetylase 3 (HDAC3), derepressed Klotho and alleviated renal fibrotic injuries in mice with unilateral ureter obstruction and aristolochic acid nephropathy 27. HDAC3 overexpression or inhibition in renal epithelia was inversely related to Klotho levels, and HDAC3 was inducibly associated with transcriptional regulators NCoR and NF-κB and bound to Klotho promoter in fibrotic kidney, reinforcing that aberrant HDAC3 targets Klotho and inhibits its transcription in renal fibrosis 27. Diabetic nephropathy is also associated with decreased renal klotho expression and renal mitochondrial injury, and klotho exerts a mitochondrial effect on renal mitochondria via LKB1-AMPK-PGC1α expression and ameliorates diabetic nephropathy 28. Moreover, Klotho alleviates kidney and cardiac injury by inactivating NF-κB signaling and promoting macrophage M2 polarization, potentially mitigating indoxyl sulfate-induced heart failure and chronic kidney disease or acute kidney injury 29. Thus, Klotho is closely associated with inflammation, renal fibrosis, and kidney disease.

Nuclear factor erythroid 2-like 2 (NFE2L2, also named Nrf2) is a redox sensitive transcription factor that regulates the basal and inducible expression of an array of antioxidant and detoxifying enzymes, including glutamate cysteine ligase, quinone oxidoreductase 1 and heme oxygenase-1. Under non-stimulated conditions, Nrf2 is rapidly degraded in the cytosol through ubiquitination mediated by Keap1 30, 31. The current study showed that NF-κB activation might also be required for Nrf2 signaling in response to redox sensitive or inflammatory triggers 30, 32. Sayan Ghosh and their group found that significant alterations in Nrf2-dependent cellular redox status, coupled with altered autophagy and increased apoptosis were noticed in the hippocampus of LPS-exposed mice 33. And they found that modulating the activity of Nrf2/NF-κB signaling pathway could achieve anti-inflammatory and antioxidant properties effect 33. Morover, Ulrike A Köhler et at found that loss of either Nrf2 or NF-κB/RelA had only a minor effect on liver homeostasis, but the double knockout mice spontaneously developed liver inflammation and fibrosis. So, they suggested that functional cross-talk of Nrf2 and NF-κB/RelA in hepatocytes protected the liver from necrosis, inflammation and fibrosis 34. A series of studies have pointed out that Nrf2/NF-κB signaling pathway is closely related to inflammatory response.

In the present study, we investigated whether in Klotho-deficient mice treated with BSA, the changes in intestinal microflora structure and effects on the immune system induce kidney damage and CKD.

## Material and methods

### Mouse model

α-Klotho heterozygous hypomorphic male mice (Klotho^-/+^, KL^-/+^), as previously described 35 were used in this study. A total of 20, specific-pathogen-free-grade, 6-8-week-old, male mice (Klotho^-/+^ [n = 10] and WT C57BL/6 [n = 10]; average weight = 28 ± 5 g) were purchased from Shanghai Model Organisms Company (Modelorg, Shanghai, China; License number: SCXK (Shanghai) 2019-0004). After 1 week of adaptive feeding, as described in previous reports 36, 5 and 1 mg/g body weight BSA (A-7906, Sigma-Aldrich, St. Louis, USA) was administered to all mice by intraperitoneal gavage for the first 2 days. Then, for 3 weeks, 1.5 mg/g body weight BSA was administered by gavage. This study was approved by the ethics committee of the Shanghai Geriatric Institute of Chinese Medicine (SHAGESYDW201908). All experiments conformed to the experimental animal regulations of the Ministry of Science and Technology.

### Hematoxylin-eosin staining

All fresh tissues were fixed in 4% paraformaldehyde (Sigma-Aldrich) for 30 min at room temperature. Tissues were dehydrated using an ethanol gradient, embedded in paraffin, sectioned (6 µm thickness), and dewaxed by immersion in xylene. Tissue sections were stained with hematoxylin-eosin (Sigma-Aldrich), permeabilized with xylene (Sigma-Aldrich), and mounted in neutral resin (Sigma-Aldrich) 35.

### Masson staining

The sections were washed with double-distilled water for 5 min and then stained with hematoxylin (Beyotime Biotechnology) for 5-10 min, followed by thorough rinsing with water. The sections were counterstained with Masson's ponceau acid fuchsin solution (Beyotime Biotechnology) for 6-10 min and then rinsed with 2% ice-cold aqueous acetic acid (Beyotime Biotechnology) for 5 s. The sections were differentiated for 3-5 min with 1% aqueous phosphomolybdic acid (Beyotime Biotechnology), stained by direct immersion in aniline blue for 5 min, and then washed with 0.2% aqueous glacial acetic acid (Beyotime Biotechnology) for several seconds. The stained sections were cleared, sealed, and photographed 9. The quantitative statistical method of collagen area from masson staining: Collegen area fraction (%) = (MASSON stainging collegen positive area / total area) * 100%”.

### Periodic acid-Schiff (PAS) staining

Briefly, the sections were washed with double-distilled water for 5 min and then stained with 10 g/L Periodate (Beyotime Biotechnology) for 20 min, followed by thorough rinsing with water. The sections were counterstained with Schiff solution (Beyotime Biotechnology) for 60 min and then rinsed with sulfite (Beyotime Biotechnology) for 3 times, followed by thorough rinsing with water. Next, the sections were counterstained with Methyl green solution (Beyotime Biotechnology) for 20 min, followed by thorough rinsing with water. The stained sections were cleared, sealed, and photographed.

### The quantitative real-time reverse transcription PCR (qRT-PCR) assay

Briefly, total cellular RNA was extracted using the Trizol Reagent (Invitrogen, Waltham, MA, USA) according to the manufacturer's protocol. The RNA was then reverse-transcribed into cDNA using a ReverTra Ace-α First Strand cDNA Synthesis Kit (TOYOBO, Osaka, Japan). cDNA was then amplified using quantitative real-time PCR (qPCR). QPCR was conducted using a RealPlex4 real-time PCR detection system from Eppendorf Co. Ltd (Hamburg, Germany), with the SyBR Green RealTime PCR Master Mix and detection dye (TOYOBO). QPCR amplification was performed over 40 cycles. The qPCR cycle consisted of denaturation at 95 °C for 15 s and annealing at 58 °C for 45 s. The target cDNA was evaluated by relative quantification. A comparative cycle threshold (Ct) method was used to determine the gene expression relative to a control (calibrator, 18S rRNA) and steady-state mRNA levels were reported as an n-fold difference relative to that of the calibrator. For each sample, the marker gene Ct values were normalized using the formula ΔCt = Ct_genes - Ct_18S rRNA. To determine the relative expression levels, the following formula was used: ΔΔCt = ΔCt_treated_group - ΔCt_control_group. Three biological replications were performed for each reaction. The 2-ΔΔCt method was applied to measure the relative marker expression. The internal control, 18S rRNA, served as a normalizer. The sequences of the real-time PCR primers were as follows: Kl-F: ACTACGTTCAAGTGGACACTACT; Kl-R: GATGGCAGAGAAATCAACACAGT; Nfkb1-F: ATGGCAGACGATGATCCCTAC; Nfkb1-R: TGTTGACAGTGGTATTTCTGGTG; Ifng-F: ATGAACGCTACACACTGCATC; Ifng-R: CCATCCTTTTGCCAGTTCCTC; Tnf-F: CCTGTAGCCCACGTCGTAG; Tnf-R: GGGAGTAGACAAGGTACAACCC; Il1b-F: GAAATGCCACCTTTTGACAGTG; Il1b-R: TGGATGCTCTCATCAGGACAG; Nfe2l2-F: TCTTGGAGTAAGTCGAGAAGTGT; Nfe2l2-R: GTTGAAACTGAGCGAAAAAGGC; 18S rRNA-F: CAGCCACCCGAGATTGAGCA; 18S rRNA-R: TAGTAGCGACGGGCGGTGTG.

### Kidney function parameter analysis

Plasma urea level was detected with a urea assay kit (DIUR-500; BioAssay Systems, Hayward, CA, USA). Plasma and urinary creatinine levels were detected using a creatinine assay kit (DICT-500; BioAssay Systems). Urinary albumin concentration was measured with a mouse-specific microalbuminuria ELISA kit (Albuwell M; Exocell, Philadelphia, PA, USA). All kidney function parameters were measured according to manufacturers' instructions 16.

### Gut microbiota analysis

As described in previous studies 37, 38, fresh fecal samples were collected during the last 5 days of the experiment to analyze the gut microbiota. Bacterial genomic DNA was extracted from frozen samples stored at -80 °C. The V3 and V4 regions of the 16S rRNA gene were amplified by PCR using specific bacterial primers (F primer: 5'-ACTCCTACGGGAGGCAGCA-3'; R primer: 5'-GGACTACHVGGGTWTCTAAT-3'). High-throughput pyrosequencing of the PCR products was performed on an Illumina MiSeq platform at Biomarker Technologies Co. Ltd. (China). The raw paired-end reads from the original DNA fragments were merged using FLASH32 and assigned to each sample, according to the unique barcodes. QIIME 39 (version 1.8.0) UCLUST 40 software was used based on 97% sequence similarity. The tags were clustered into operational taxonomic units. The alpha diversity index was evaluated using Mothur software (v.1.30). The diversity index was compared among samples by standardizing the number of sequences contained in each sample. Operational taxonomic unit rank curves, rarefaction curves, and Shannon curves were constructed, and Shannon, Chao1, Simpson, and abundance-based coverage estimator indexes were calculated. For beta diversity analysis, heatmaps of RDA-identified key operational taxonomic units, principal coordinate analysis 41, non-metric multidimensional scaling 42, and unweighted pair-group method with arithmetic mean were obtained using QIIME. The linear discriminant analysis-effect size (LEfSe) method was used for the quantitative analysis of biomarkers in each group. The linear discriminant analysis-effect size (linear discriminant analysis threshold >4), non-parametric factorial Kruskal-Wallis sum-rank test, and unpaired Wilcoxon rank-sum test were performed to identify the taxa with significantly different abundance 43, 44.

### Western blot

Western blot analysis of protein expression was performed according to a previously described method 35. In brief, total protein lysates were subjected to 12% sodium dodecyl sulfate polyacrylamide gel electrophoresis and transferred onto hybrid polyvinylidene fluoride membranes (Millipore, Bedford, USA). After blocking, the polyvinylidene fluoride membranes were washed four times for 15 min with Tris-buffered saline containing Tween-20 at room temperature, followed by incubation with the primary antibody (Table S2). Following extensive washing, membranes were incubated with the secondary antibody (Table S2) for 1 h. After washing, the immunoreactivity was visualized by enhanced chemiluminescence using an ECL kit (Perkin-Elmer Life Science, Norwalk, USA).

### Superoxide dismutase assay

The superoxide dismutase assay was performed using the SOD activity assay kit (Beyotime, Shanghai, China) according to the manufacturer's instructions 35. In brief, 200 µL of the sample lysate was added to 1 × 10^6^ cells/mL and was thoroughly mixed by pipetting. The mixture was centrifuged at 12,000 × *g* for 5 min at 4°C, and the supernatant was collected. The WST-8/enzyme working solution was prepared by thoroughly mixing 151 µL of superoxide dismutase assay buffer, 8 µL of WST-8, and 1 µL of enzyme solution. A concentration gradient of superoxide dismutase standard solutions was prepared (100 U/mL, 50 U/mL, 20 U/mL, 10 U/mL, 5 U/mL, 2 U/mL, and 1 U/mL) and tested simultaneously with the samples. Twenty microlitres of the cell lysis supernatant and standard solutions were added to 160 µL of freshly prepared WST-8/enzyme working solution and 20 µL of reaction initiation solution, respectively. After thorough mixing, the samples were incubated at 37 °C for 30 min. The absorbance was measured at 450 nm.

### Flow cytometry analysis

Mouse peripheral blood mononuclear cells (PBMCs) from each group were suspended (1 × 10^6^ cells/mL) and stained with the primary antibodies (Table S2) on ice in Dulbecco's phosphate-buffered saline containing 10% BSA. Staining with an isotype control antibody (mouse IgG1-FITC, mouse IgG1-PE; Invitrogen, eBioscience™, Shanghai, China) was used to correct for non-specific binding. Antibody staining was analyzed by fluorescence correlation microscopy using a FACS Aria (Quanta SC, Beckman Coulter INC) 35.

### Statistical analysis

Each experiment was performed as least thrice; data are expressed as mean ± standard error where applicable; differences were evaluated with Student's t-test. A *P* value <0.05 was considered statistically significant.

## Results

### Bovine serum albumin caused significantly more kidney damage in Kl^-/+^ mice than in WT mice

**Figure 1:**
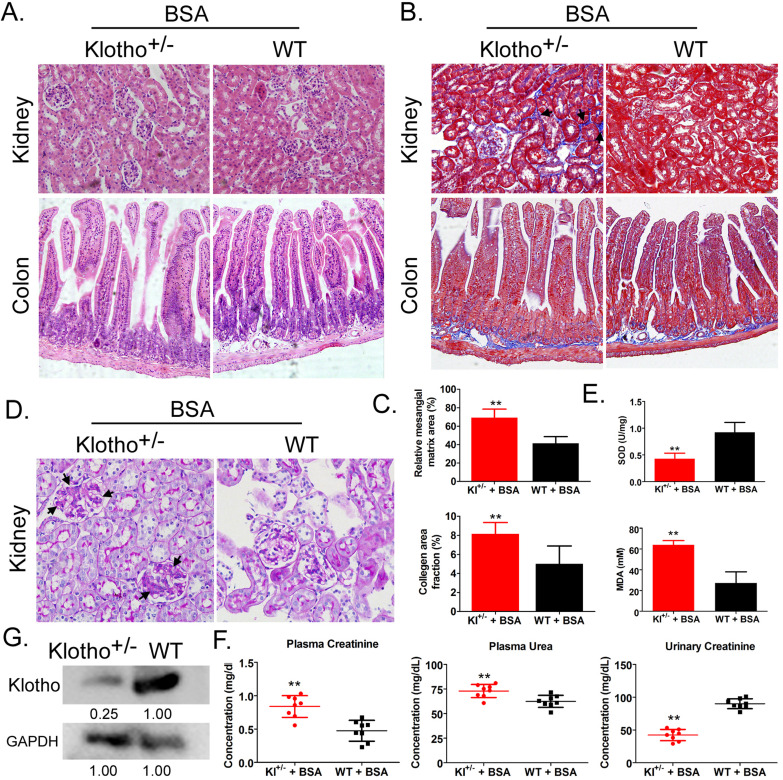
** Bovine serum albumin (BSA)-induced kidney injury in mice.** (A) Hematoxylin and eosin staining. Magnification 200×. (B) Masson staining. Magnification 200×. The arrow indicated the collagen deposition sites. (C) Comparisons of mesangial matrix area and collagen area fraction of kidney tissues in BSA-treated Klotho-deficient and wild-type mice. **p < 0.01 (t test); n = 4. (D) Periodic acid-Schiff staining results. Magnification 200×. The arrow indicated the mesangial hyperplasia sites. (E) Results of serum superoxide dismutase and malondialdehyde assays of BSA-treated Klotho-deficient and wild-type mice. **p < 0.01 (t test); n = 4. (F) Creatinine and urea in serum and urea in urine in BSA-treated Klotho-deficient and wild-type mice. **p < 0.01 (t test); n = 4. (G) Western blot of Klotho in the kidney tissue of BSA-treated Klotho-deficient and wild-type mice.

Hemoxylin-eosin, Masson, and periodic acid-Schiff staining showed that after BSA treatment, Kl^-/+^ mice (BSA-Kl^-/+^) showed significantly higher renal mesangial proliferation, and significantly larger area of glomerular collagen deposition, and fibrosis than WT mice (BSA-WT) (Fig. 1A-D). Moreover, the peripheral blood levels of superoxide dismutase (SOD) in BSA-Kl^-/+^ mice were significantly lower than it in BSA-WT mice (Fig. 1E). However, the peripheral blood levels of malondialdehyde (MDA) in BSA-Kl^-/+^ mice were significantly higher than those in BSA-WT mice (Fig. 1E). In addition, BSA-Kl^-/+^ mice significantly higher levels of peripheral blood creatinine and urea and significantly lower levels of urinary creatinine than BSA-WT mice (Fig. 1F). Western blot showed that Klotho expression in the kidney was significantly lower in BSA-Kl^-/+^ mice than in BSA-WT mice (Fig. 1G). The results indicate that, after BSA treatment, Kl^-/+^ mice showed a significantly greater degree of kidney damage than WT mice.

### Bovine serum albumin disrupts the balance of intestinal bacteria distribution and metabolism in Kl^-/+^ mice

**Figure 2:**
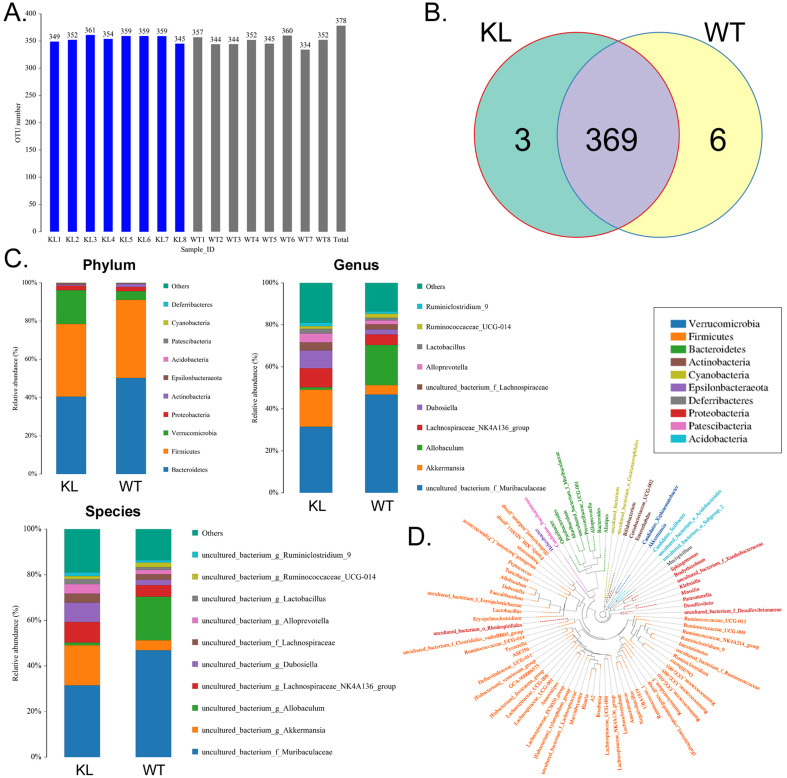
** Analysis of operational taxonomic units.** (A) Number of operational taxonomic units. (B) Venn diagram of operational taxonomic units. (C) Gut microbiota clustering and species distribution. (D) Species phylogenetic tree.

The fecal samples of BSA-treated Kl^-/+^ mice and WT mice were processed and used for high-throughput sequencing of bacterial 16s rRNA v3-v4 regions to assess the specific composition and distribution of gut microbiota. Sequencing of 16 samples yielded 1,259,070 paired reads, producing 1152150 clean tags after joining and filtering the reads. Each sample produced at least 63,287 clean tags, producing an average of 72,009 clean tags (Table S1). Using QIIME (version 1.8.0) UCLUST software, tags were clustered into operational taxonomic units based on 97% similarity. The Kl^-/+^ and WT groups showed no significant difference in the number of operational taxonomic units (Fig. 2A). They had 369 operational taxonomic units overlapping; the Kl^-/+^ group showed three unique operational taxonomic units, whereas WT has six unique operational taxonomic units (Fig. 2B). By comparing the sequence of the operational taxonomic units to a microbial reference database, each operational taxonomic unit can be assigned to a species. The community composition of each sample was also characterized. QIIME software was used to generate the species abundance at different levels of classification (Kingdom, Phylum, Class, Order, Family, Genus, and Species). Using R language tools, the community structure of the samples at different classification levels was determined. Phylum-level analysis showed that the Kl^-/+^ group mice showed a significantly higher relative abundance of phylum Verrucomicrobia and significantly lower relative abundance of phylum Bacteroidetes than the control group mice.

**Figure 3:**
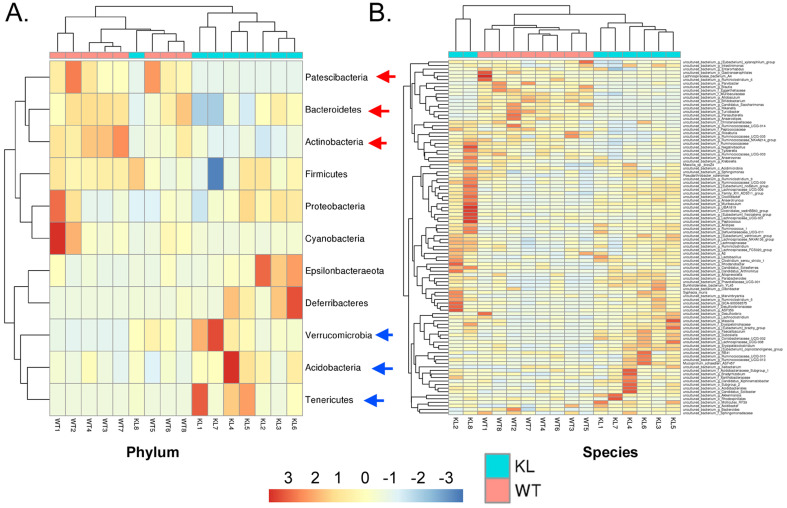
Heat map of species richness clustering at the level of: (A) Phylum; (B) Species.

Genus-level analysis showed that the Kl^-/+^ group mice showed a significantly higher relative abundance of the genera *Dubosiella*, *Akkermansia*, *Alloprevotella*, and *Lachnospiraceae* and significantly lower relative abundance of the genera *Allobaculum* and *Muribaculaceae* than the control group mice.Species-level analysis showed that the Kl^-/+^ group mice showed a significantly higher relative abundance of species belonging to the genera *Lachnospiraceae*, *Dubosiella*, *Alloprevotella*, *Akkermansia* microbes increased significantly, and a significantly lower relative abundance of species belonging to the genera *Allobaculum* and *Muribaculaceae* than the control group mice (Fig. 2C-D). Cluster analysis showed that the intestinal microbial diversity of the superphylum Patescibacteria, especially the phyla Bacteroidetes and Actinobacteria, was significantly lower and that of the phyla Verrucomicrobia, Acidobacteria, and Tenericutes was significantly higher in the Kl^-/+^ group mice than in the control group mice (Fig. 3).

**Figure 4:**
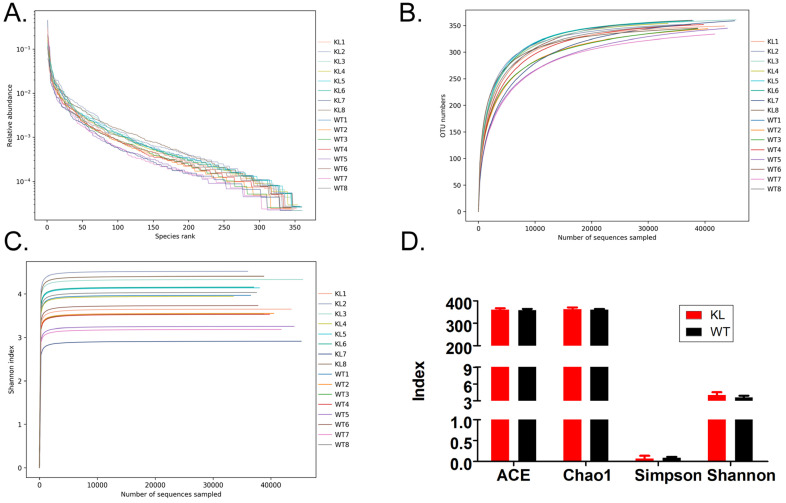
** Alpha diversity analysis.** (A) Rank abundance curve; (B) Rarefaction curve; (C) Shannon index curve; (D) Alpha diversity analysis.

**Figure 5:**
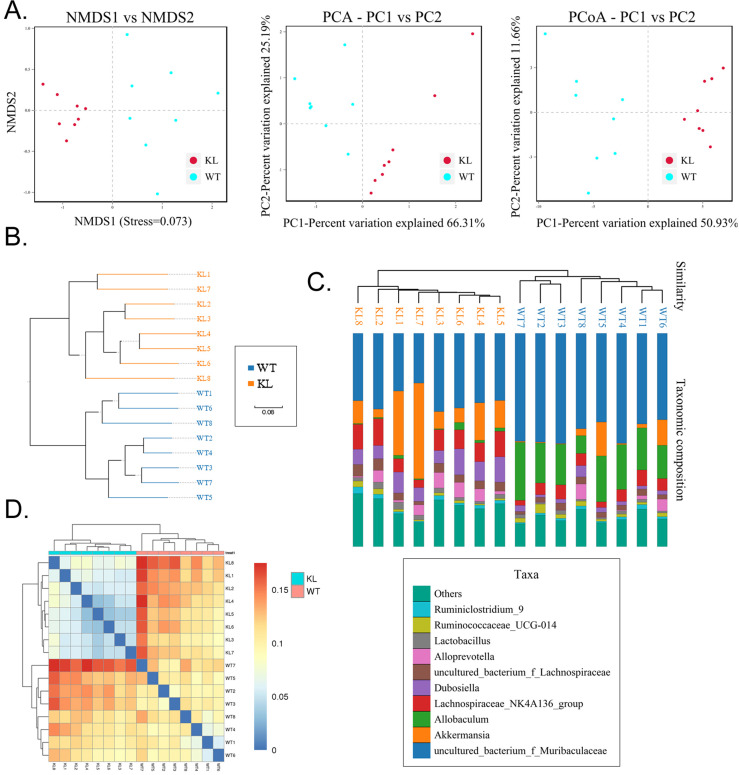
** Beta diversity analysis.** (A) Results of principal component analysis, principal coordinate analysis, and non-metric multidimensional scaling analysis. (B) Sample unweighted pair-group method with arithmetic mean clustering tree. (C) Combined drawing of clustering tree and histogram. (D) Heat map showing sample abundance.

Alpha diversity analysis found that rank-abundance curve showed a flat curve, suggesting a highly homogeneous species composition (Fig. 4A); Shannon curves tended to be flat, indicating that sequencing data quantity was sufficiently large; thus, the number of operational taxonomic unit species does not increase with the increasing quantity of sequencing data (Fig. 4B). Rarefaction curves were smooth, indicating that sample sequences were complete and data analysis could be performed (Fig. 4C). Beta diversity analysis using the Bray-Curtis algorithms found differences in the microbial flora between groups. The analysis mainly includes the principal coordinate analysis, principal component analysis, and non-metric multidimensional scaling. According to the above analyses, microbial communities in the Kl^-/+^ and WT group samples show obvious differences of the distribution of group manager namely Kl^-/+^ group of microorganisms and WT group shall form a separate community (Fig. 5A). Unweighted pair-group method with arithmetic mean sample hierarchy clustering analysis results suggest that Kl^-/+^ and WT groups show a low homology of the gut microbiota, and there was no close genetic association (Fig. 5B-5D).

**Figure 6:**
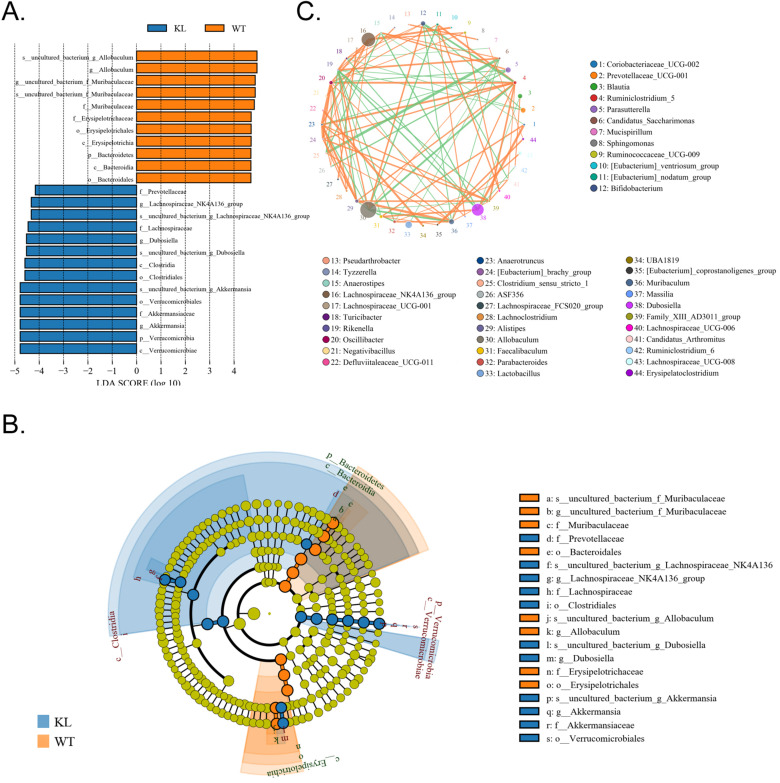
** Significant difference analysis between groups.** (A) Value distribution histogram of line discriminant analysis effect size. (B) Results of species annotation were visualized using KRONA. (C) Species network at the genus level.

In addition, the line discriminant analysis (LDA) effect size was used to identify each species of the gut microbiota as important biomarkers; species of LDA scores >4 are considered to be important biological markers. Cladogram analysis and LDA score distribution indicate that in the Kl^-/+^ group, a significantly high number of microorganisms belonging to phylum Verrucomicrobia, class Verrucomicrobiae, order Verrucomicrobiales, family *Akkermansiaceae*, and genus *Akkermansia;* therefore, the unique advantages of microbial community is Kl^-/+^ group strains (Fig. 6A-C).

### Functional genes of gut microbes in Kl^-/+^ mice and the difference between the expression of metabolic pathway

**Figure 7:**
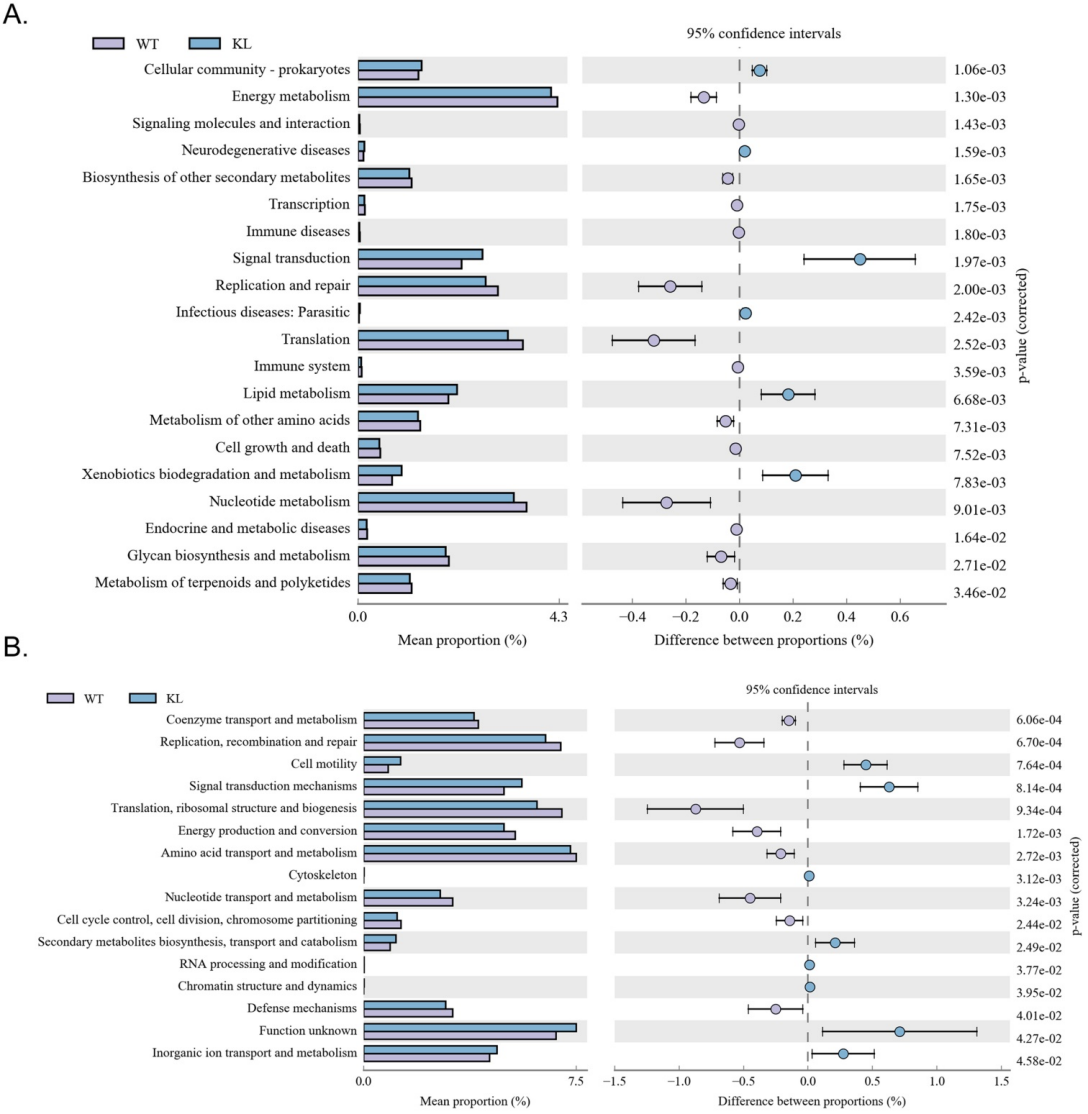
** Metabolic signaling pathways and protein differences in gut microbiota.** (A) Results of Kyoto Encyclopedia of Genes and Genomes metabolic pathway analysis. (B) Clusters of orthologous groups of proteins analysis of distribution and abundance of homologous protein clusters in gut microbiota.

Kyoto Encyclopedia of Genes and Genomes metabolic pathway analysis showed differences between samples from different groups in microbial community functional genes in metabolic pathways so as to explore the metabolic function change in different samples to adapt to environmental changes of (Table S5). Comparison of the KI^-/+^ group with the WT group showed that the intestinal microbes of the Kl^-/+^ group have functional genes contributing to metabolic pathways in signal transduction (environmental information processing), xenobiotic biodegradation and metabolism (metabolism), lipid metabolism (metabolism), translation-based information processing, and nucleotide metabolism-based information processing (such as replication and repair; Fig. 7A).

Analysis of clusters of orthologous groups of proteins showed that the distribution of the microbial homologous protein cluster belong to spend (Table S5). Results showed that the abundance of intestinal microbial proteins involved in metabolic pathways related signal transduction mechanisms (cellular the processes and signaling) and cell motility (cellular the processes and signaling) was significantly higher and that of intestinal microbial proteins involved in ribosomal structure and biogenesis (information storage and processing); replication, recombination, and repair (information storage and processing) was significantly lower in the Kl^-/+^ group than in the control group (Fig. 7B).

### Bovine serum albumin treatment leads to unusually high number and activity of MΦ1 in Kl^-/+^ mice

**Figure 8:**
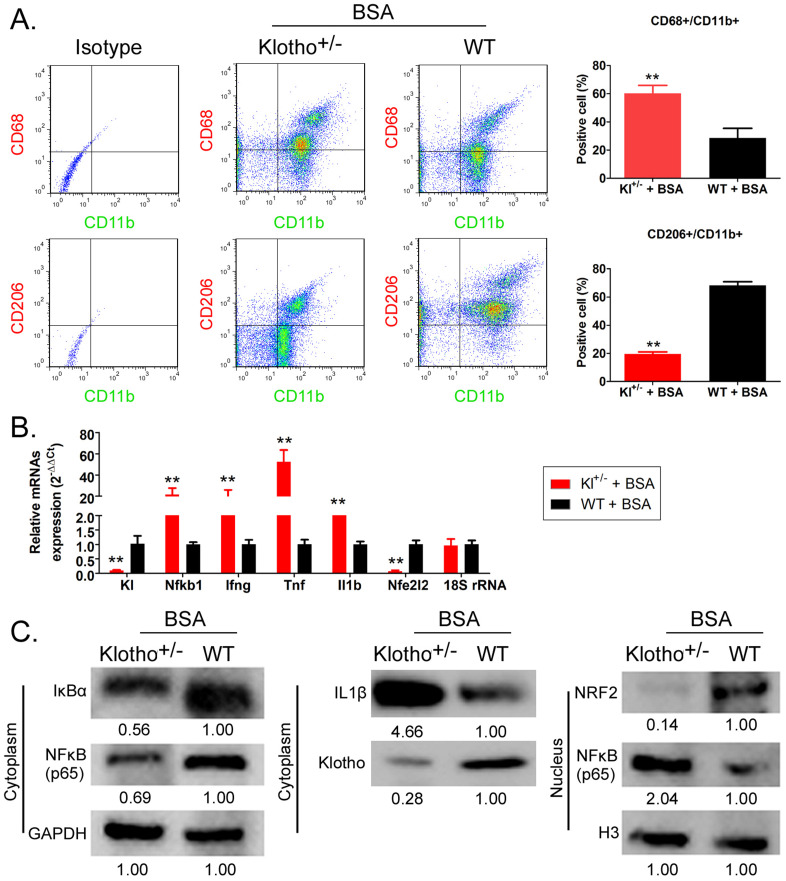
** Bovine serum albumin (BSA) increased the number and activity of MΦ1.** (A) Analysis of the proportion of monocyte-derived macrophage subsets using flow cytometry in BSA-treated Klotho-deficient and wild-type mice. **p < 0.01 (t test); n = 4. (B) qPCR results **p < 0.01 vs WT+BSA; t test; n = 4. (C) Western blot results.

Flow cytometry results showed that the proportion of CD68^+^/CD11b^+^ cells (the source of mononuclear MΦ1 cells) in peripheral blood was significantly higher in BSA-Kl^-/+^ mice than that in BSA-WT mice; however, in the peripheral blood of, the proportion of CD206^+^/CD11b^+^ cells (the source of mononuclear MΦ2 cells) was significantly lower in BSA-Kl^-/+^ mice than that in BSA-WT mice significantly (Fig. 8A). qPCR results indicated that *KL* (Klotho) and *Nfe2l2* (*-*Nrf2) gene expression level in the peripheral blood cells was significantly lower in BSA-Kl^-/+^ mice than in BSA-WT mice (Fig. 8B). However, the expression level of inflammation-related genes *Nfkb1* (NFκB) predominate, *Ifng* (IFNγ), *Tnf* (TNFα), and *Il1b* (IL-1β) in peripheral blood cells (MΦ1) in BSA-Kl^-/+^ mice was significantly higher than those in BSA-WT mice (Fig 8B). Similarly, western blot results indicated the higher expressions of IL-1β and NFκB (p65) and lower expressions of Klotho and Nrf2 in peripheral blood cells (MΦ1) from BSA-Kl^-/+^ mice, which prompts MΦ1 activation (Fig. 8C). These findings suggest that BSA treatment in Kl^-/+^ mice activates MΦ1 cells by regulating Nrf2/NF-κB signaling pathways.

## Discussion

The intestine - the main organ of uremic toxin production and discharge 1,4-8,10 - exhibits mechanical, mucous, immune, and biological barriers, which maintain the intestinal tract and the internal environment of the body in a steady state 1,4-8,10. Among them, the biological barrier is mainly composed of a large number of bacteria, viruses, protozoa, and fungi that parasitize the surface of the intestinal lumen 1, 4-8, 10. Under normal circumstances, the intestinal microflora and the body are in dynamic equilibrium; when this balance is disrupted, it may damage the function of the intestinal barrier, leading to a change in the number and proportion of gut bacteria, or translocate them, causing endogenous infection 1,4-8,10. Some uremic toxins (such as AGE, phenols, indoles and TMAO) have strong biological effects 1,4-8,10; Mishima and others analyzed the distribution and structure of approximately 190 types of intestinal microorganisms in patients with renal failure and found an imbalance in their abundance; specifically, the abundance of lactic acid bacteria was significantly lower and that of fecal bacteria, *Escherichia coli*, and other microorganisms was significantly higher 40. An imbalance in the intestinal microecology--a decrease in the number and diversity of intestinal probiotics and an increase in the number and diversity of pathogens-damages the intestinal mucosa barrier, increases the intestinal mucosal permeability, translocates pathogenic bacteria and enterogenous endotoxins into the blood circulation, activates the intestinal mucosal immune system, and induces systemic inflammatory reaction, causing kidney damage. The increase in the number of pathogenic bacteria produce urinary toxin, intestinal source of cresol sulfate, sulfuric acid indole phenol, trimethylamine oxide accumulation in the blood, and kidney function impairment and fail to eliminate the toxin, resulting in further kidney damage and function impairment. Whibley and others found that after *Candida albicans* transplantation, the disruption of the intestinal flora in mice induced a significant rise in number of Treg cells, thus disrupting the immune balance.

Simultaneously, an increase in the intestinal pathogenic bacteria activates kidney through its metabolites inside and outside of the macrophage recognition receptors immune response, thus causing kidney damage 41. Therefore, intestinal dysbacteriosis causes kidney damage through effects on the immune system, release of urinary toxins, and translocation of pathogenic bacteria translocation 1, 4-8, 10, 40, 41. The expression of longevity-related genes such as Klotho is also closely associated with renal fibrosis, chronic kidney disease, and immune system imbalance [11, 23, 29, 42 to 45]. Blood vessel endothelial cell aging and atherosclerosis, glomerular fibrosis and kidney damage, and skeletal muscle loss significantly increases the risk of degenerative diseases in Klotho-deficient mice [11,23,29,42 to 45]. In addition, Klotho expression maintains the activity of CD4^+^ T cells (Th1), and reduction in Klotho expression significantly induces CD4^+^ T cell aging and death 46. Klotho also promotes Macrophage M1-to-M2 transformation, thus inducing damage repair 47. In absence of Klotho expression, an imbalance in FGF23/FGFR/α-Klotho signaling pathway severely affects the Mφ1-to-Mφ2 transformation, leading to an accumulation of inflammatory Mφ1 cells, eventually inducing damage owing to chronic inflammation 47. Intestinal dysbacteriosis and abnormal immune system cause kidney damage and CKD. Therefore, the present study aimed to explore whether there is an association between the intestinal flora, Klotho, and inflammatory cells, especially macrophages and whether they jointly maintain the kidney function and homeostasis.

To answer the above questions, we selected Klotho-deficient heterozygous mice as the research object. We used small doses of BSA (the dose of BSA reported to induce kidney damage in mice is approximately 10-15 mg/g 31, but we selected a dose 1/10 of this dose) to achieve drug-induced kidney injury in mice to investigate whether Klotho mice are more prone to drug-induced kidney damage than WT mice. Histopathological staining and creatinine and urea levels suggest that BSA treatment of Klotho-deficient mice by intraperitoneal gavage causes glomerular fibrosis and mesangial proliferation. The abnormal increase in serum creatinine and urea in Klotho-deficient mice also induce kidney damage; however, the changes were insignificant in WT mice. The results indicate that in absence of Klotho expression in mice, small doses of albumin stimulation for a long time keep burdening the kidney, causing kidney damage.

We also analyzed the distribution, quantity, and community structure of intestinal bacterial flora and the difference in metabonomics in both groups of mice. 16s rRNA v3-v4 sequencing indicated that the intestinal flora of the two groups of mice exhibits significant differences. After long time feeding BSA, the intestinal flora in Klotho-deficient mice treated with BSA for long periods exhibit a significant increase in the relative abundance of microbial species of the genera *Dubosiella*, *Akkermansia*, *Alloprevotella*, *Lachnospiraceae*, and *Muribaculaceae* and significant reduction in the relative abundance of microorganisms belonging to the genus *Allobaculum*.

Microbial diversity and cluster analysis indicated that the number and microbial diversity of the superphylum Patescibacteria, especially the phyla Bacteroidetes and Actinobacteria, decreased and, and the number of microorganisms in the phyla Verrucomicrobia, Acidobacteria, and Tenericutes increased in BSA-treated Klotho-deficient mice. A significant change in the intestinal microecological structure eventually led to a significant change in the metabolites produced by the intestinal microorganism. In BSA-treated Klotho-deficient mice, metabolic pathways associated with signal transduction, xenobiotics biodegradation and metabolism, lipid metabolism substances were significantly more active than that in the control group; the abundance of proteins involved in signal transduction mechanisms and cell motility also improved significantly. Our previous studies 9, 48 suggest that a change in gut microbial species stimulates the intestinal mucosal barrier and mucosal immune changes, affecting the distribution of intestinal immune cells, immune cells, and peripheral blood and polarity of anomalies.

Stanford and others summarized several reports on the intestinal flora of patients with kidney disease and pointed out that the abundance of several microorganisms (including Enterobacteriaceae and *Streptococcus*) is significantly higher in adult patients with kidney disease, specifically, the difference in the abundance of genus *Lachnospiraceae* was very significant between groups 45. Moreover, Li and colleagues also reported that the abundance of genera *Lactobacillus*, *Clostridium* IV, *Paraprevotella*, *Clostridium* (sensu stricto), *Desulfovibrio*, and *Alloprevotella* was higher in the fecal samples of patients with chronic kidney disease than that in the healthy control group 46. The reports are consistent with the findings of our study; this shows that kidney damage induced by BSA intervention and chronic kidney disease in mice is similar to intestinal flora disorder or CKD in humans, and the strain distribution and community structure of intestinal microorganisms is very similar to that in humans. Our findings suggested that in patients with CKD, intestinal microorganisms of the genera *Alloprevotella* and *Lachnospiraceae* are more abundant. Imbalance of intestinal flora will accompany the immune system and inflammatory cells proportion of exceptions, the more has been written. The results also suggest that BSA treatment in mice increases the number of macrophages, especially MΦ1. However, BSA-treated Klotho-deficient mice showed a disrupted ratio of peripheral blood MΦ1 and MΦ2; the number of MΦ1 continued to increase, and that of MΦ2 continued to decline. This finding suggests that Klotho deficiency induces persistent inflammation (MΦ1 cause inflammation), and significantly impairs the tissue repair ability (MΦ2 have tissue repair function). BSA-treated Klotho-deficient mice also showed higher expression of NFκB (p65) and lower expression of Nrf2 protein in MΦ1 cells, prompting MΦ1 activation. This result is consistent with previously reported findings 51.

**Figure 9:**
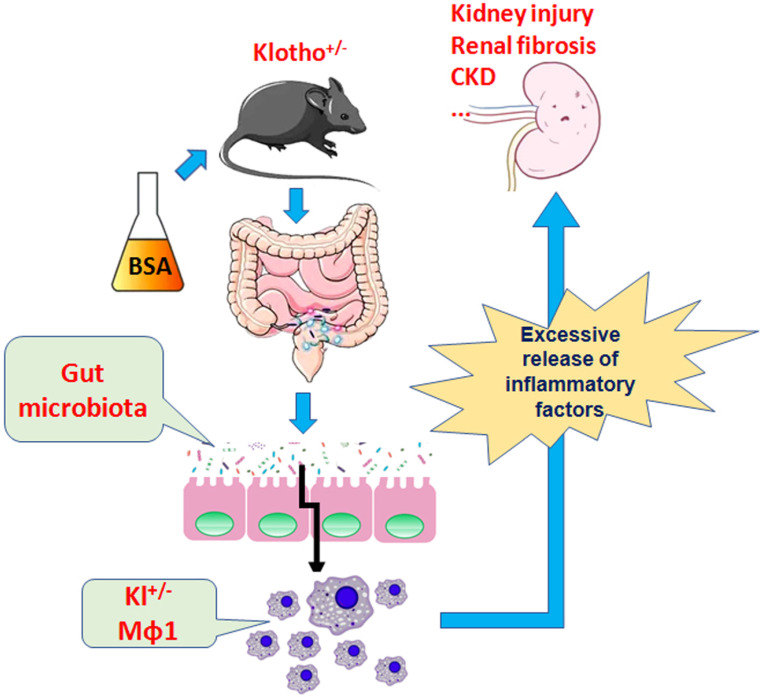
BSA aggravates macrophage M1 activation and kidney injury in heterozygous Klotho-deficient mice via the gut microbiota-immune axis.

Therefore, the present study demonstrates that BSA alters the community structure of the intestinal flora and induces kidney damage and chronic kidney disease in mice and promotes immune activation and chronic inflammation in Klotho-deficient mice with intestinal dysbacteriosis (Fig 9). Changes in the number of immune cells, especially the source of mononuclear macrophages and imbalance in the expression of proteins in the Nrf2/NFκB signaling pathway lead to increased inflammation and kidney damage.

## Supplementary Material

Supplementary figures and tables.
