# Supplementary material for: Bovine serum albumin aggravates macrophage M1 activation and kidney injury in heterozygous Klotho-deficient mice via the gut microbiota-immune axis
Source: Int J Biol Sci. 2021 Feb 2;17(3):742–55. doi: 10.7150/ijbs.56424 (PMC7975693; doi:10.7150/ijbs.56424)
Supplement: Supplementary file 1 — Supplementary figures and tables. [file ijbsv17p0742s1.pdf]

Table S1. Primary antibodies.

Antibodies	Companies	Applications
Rabbit anti-Klotho antibody [EPR6856] (ab181373)	Abcam, MA, USA	WB (1:1000)
NRF2 (D1Z9C) XP Rabbit mAb (#12721)	Cell Signaling Technology, MA, USA	WB (1:1000)
IκBα (44D4) Rabbit mAb (#4812)	Cell Signaling Technology, MA, USA	WB (1:1000)
NF-κB p65 Antibody Sampler Kit (#4767)	Cell Signaling Technology, MA, USA	WB (1:1000)
Rabbit anti-GAPDH antibody [EPR16891] (ab181602)	Abcam, MA, USA	WB (1:1000)
Goat anti-Rabbit IgG H&L (HRP) (ab97051)	Abcam, MA, USA	WB (1:1000)
Goat anti-Mouse IgG H&L (HRP) (ab6789)	Abcam, MA, USA	WB (1:1000)
Histone H3 (D1H2) XP Rabbit mAb (#4499)	Cell Signaling Technology, MA, USA	WB (1:1000)
CD68 (D4B9C) XP Rabbit mAb (PE Conjugate) (#79594)	Cell Signaling Technology, MA, USA	FCM (1:200)
CD11b/ITGAM (M1/70) Rat mAb (FITC Conjugate)	Cell Signaling Technology, MA, USA	FCM (1:200)

(#24442)		
Anti-CD206/Mannose Receptor antibody [EPR6828(B)] (PE) (ab223960)	Abcam, MA, USA	FCM (1:200)

Table S2 Statistical table of per level Tags of samples

Sample	Kindom	Phylum	Class	Order	Family	Genus	Species
KL1	43,537	43,537	43,537	43,537	43,529	43,537	43,537
KL2	36,022	36,022	36,022	36,022	36,018	36,022	36,022
KL3	45,630	45,630	45,630	45,630	45,626	45,630	45,630
KL4	33,748	33,748	33,748	33,748	33,743	33,748	33,748
KL5	38,073	38,073	38,073	38,073	38,068	38,073	38,073
KL6	37,058	37,058	37,058	37,058	37,054	37,058	37,058
KL7	45,309	45,309	45,309	45,309	45,304	45,309	45,309
KL8	38,907	38,907	38,907	38,907	38,902	38,907	38,907
WT1	36,561	36,561	36,561	36,561	36,559	36,561	36,561
WT2	40,715	40,715	40,715	40,715	40,710	40,715	40,715
WT3	38,799	38,799	38,799	38,799	38,792	38,799	38,799

WT4	39,925	39,925	39,925	39,925	39,921	39,925	39,925
WT5	44,243	44,243	44,243	44,243	44,240	44,243	44,243
WT6	37,891	37,891	37,891	37,891	37,888	37,891	37,891
WT7	41,965	41,965	41,965	41,965	41,963	41,965	41,965
WT8	37,678	37,678	37,678	37,678	37,676	37,678	37,678

Table S3 Alpha diversity index statistics

Sample ID	OTU	ACE	Chao1	Simpson	Shannon	Coverage
KL1	349	353.2136	355.0667	0.1064	3.6512	0.9997
KL2	352	358.9083	360.0	0.0203	4.5188	0.9995
KL3	361	365.941	371.9091	0.0282	4.3332	0.9996
KL4	354	359.1358	358.375	0.0572	3.9432	0.9996
KL5	359	363.6152	367.0769	0.0432	4.1403	0.9996
KL6	359	363.09	364.0556	0.0367	4.1572	0.9996
KL7	359	371.4507	375.6071	0.2164	2.9113	0.9993
KL8	345	352.0847	354.5625	0.0287	4.4072	0.9995
WT1	357	360.1189	359.619	0.062	3.9666	0.9997
WT2	344	357.4356	359.6154	0.085	3.5518	0.9993
WT3	344	363.4491	363.3448	0.0891	3.5345	0.9991

WT4	352	358.419	361.5	0.0904	3.5304	0.9995
WT5	345	363.5957	362.0	0.1015	3.2507	0.9992
WT6	360	364.5413	363.7917	0.0641	3.7336	0.9996
WT7	334	347.6667	353.84	0.1216	3.1857	0.9992
WT8	352	359.6544	363.6667	0.0442	4.0327	0.9994

Table S4 Analysis of KEGG metabolic pathway

Class1	Class2	WT: mean rel.freq.(%)	WT: std.dev.(%)	KL: mean rel.freq.(%)	KL: std.dev.(%)	p-values (corrected)	Difference between means
Genetic Information Processing	Translation	3.529	0.124	3.208	0.143	0.003	0.320
Metabolism	Nucleotide metabolism	3.609	0.114	3.336	0.164	0.009	0.273
Genetic Information Processing	Replication and repair	2.990	0.098	2.731	0.108	0.002	0.259
Metabolism	Energy metabolism	4.267	0.043	4.133	0.039	0.001	0.134
Metabolism	Glycan	1.947	0.036	1.878	0.050	0.027	0.070

	biosynthesis and metabolism						
Metabolism	Amino acid metabolism	6.874	0.031	6.819	0.065	0.116	0.055
Metabolism	Metabolism of other amino acids	1.338	0.023	1.285	0.030	0.007	0.053
Metabolism	Biosynthesis of other secondary metabolites	1.147	0.017	1.103	0.016	0.002	0.044
Metabolism	Metabolism of terpenoids and polyketides	1.149	0.020	1.114	0.026	0.035	0.035
Metabolism	Carbohydrate	9.889	0.067	9.857	0.158	0.726	0.032

	metabolism						
Metabolism	Metabolism of cofactors and vitamins	3.951	0.020	3.930	0.024	0.142	0.021
Cellular Processes	Cell growth and death	0.475	0.007	0.460	0.008	0.008	0.015
Cellular Processes	Transport and catabolism	0.499	0.013	0.484	0.012	0.085	0.015
Human Diseases	Endocrine and metabolic diseases	0.199	0.006	0.187	0.008	0.016	0.012
Genetic Information Processing	Transcription	0.146	0.004	0.136	0.003	0.002	0.010
Organismal Systems	Nervous system	0.224	0.007	0.215	0.010	0.115	0.009

Organismal Systems	Immune system	0.082	0.002	0.075	0.004	0.004	0.007
Environmental Information Processing	Signaling molecules and interaction	0.042	0.001	0.037	0.002	0.001	0.004
Human Diseases	Immune diseases	0.041	0.002	0.037	0.001	0.002	0.004
Organismal Systems	Digestive system	0.026	0.004	0.022	0.006	0.231	0.004
Human Diseases	Infectious diseases: Bacterial	0.396	0.002	0.393	0.004	0.089	0.003
Human Diseases	Cancers: Specific types	0.056	0.003	0.053	0.003	0.139	0.003
Organismal Systems	Environmental adaptation	0.145	0.003	0.144	0.007	0.906	0.001
Human Diseases	Drug resistance:	0.002	0.000	0.002	0.000	1.005	0.000

	Antineoplastic						
Organismal Systems	Sensory system	0.000	0.000	0.000	0.000	1.000	0.000
Cellular Processes	Cellular community - eukaryotes	0.000	0.000	0.000	0.000	1.022	0.000
Organismal Systems	Development	0.000	0.000	0.000	0.000	1.045	0.000
Human Diseases	Infectious diseases: Viral	0.001	0.000	0.001	0.000	0.821	0.000
Human Diseases	Cardiovascular diseases	0.000	0.000	0.001	0.000	0.139	0.000
Organismal Systems	Circulatory system	0.001	0.000	0.002	0.001	0.078	-0.001
Human Diseases	Cancers: Overview	0.547	0.008	0.552	0.016	0.567	-0.005
Organismal Systems	Endocrine system	0.556	0.008	0.562	0.014	0.506	-0.006
Organismal Systems	Aging	0.316	0.009	0.324	0.020	0.465	-0.008

Genetic Information Processing	Folding, sorting and degradation	1.354	0.021	1.365	0.041	0.659	-0.011
Organismal Systems	Excretory system	0.030	0.007	0.040	0.013	0.121	-0.011
Human Diseases	Drug resistance: Antimicrobial	0.849	0.012	0.864	0.021	0.176	-0.015
Human Diseases	Substance dependence	0.009	0.009	0.027	0.016	0.056	-0.018
Human Diseases	Neurodegenerative diseases	0.117	0.006	0.136	0.006	0.002	-0.018
Human Diseases	Infectious diseases: Parasitic	0.025	0.007	0.047	0.010	0.002	-0.022
Metabolism	Global and overview maps	43.128	0.109	43.184	0.230	0.680	-0.056

Cellular Processes	Cellular community - prokaryotes	1.292	0.026	1.366	0.020	0.001	-0.074
Environmental Information Processing	Membrane transport	3.363	0.076	3.487	0.145	0.119	-0.124
Cellular Processes	Cell motility	0.506	0.110	0.682	0.159	0.068	-0.176
Metabolism	Lipid metabolism	1.937	0.071	2.119	0.100	0.007	-0.181
Metabolism	Xenobiotics biodegradation and metabolism	0.728	0.087	0.937	0.121	0.008	-0.209
Environmental Information Processing	Signal transduction	2.217	0.177	2.666	0.184	0.002	-0.449

Table S5 Analysis of clusters of orthologous groups of proteins function prediction

Class1	Class2	WT: mean rel.freq.(%)	WT: std.dev.(%)	KL: mean rel.freq.(%)	KL: std.dev.(%)	p-values (corrected)	Difference between means
INFORMATION STORAGE AND PROCESSING	Translation, ribosomal structure and biogenesis	6.992	0.337	6.119	0.311	0.001	0.873
INFORMATION STORAGE AND PROCESSING	Replication, recombination and repair	6.950	0.193	6.419	0.124	0.001	0.531
METABOLISM	Nucleotide transport and metabolism	3.150	0.189	2.699	0.223	0.003	0.451
METABOLISM	Energy production and	5.354	0.164	4.957	0.159	0.002	0.397

	conversion						
CELLULAR PROCESSES AND SIGNALING	Defense mechanisms	3.143	0.131	2.892	0.220	0.040	0.251
METABOLISM	Amino acid transport and metabolism	7.505	0.101	7.292	0.082	0.003	0.213
METABOLISM	Coenzyme transport and metabolism	4.050	0.044	3.900	0.047	0.001	0.150
CELLULAR PROCESSES AND SIGNALING	Cell cycle control, cell division, chromosome partitioning	1.320	0.067	1.179	0.105	0.024	0.142
CELLULAR PROCESSES	Cell wall/membrane/envelope	7.692	0.144	7.556	0.298	0.360	0.136

AND SIGNALING	biogenesis						
CELLULAR PROCESSES AND SIGNALING	Posttranslational modification, protein turnover, chaperones	3.328	0.052	3.253	0.108	0.164	0.074
CELLULAR PROCESSES AND SIGNALING	Nuclear structure	0.000	0.000	0.000	0.000	1.000	0.000
CELLULAR PROCESSES AND SIGNALING	Extracellular structures	0.000	0.000	0.000	0.000	1.042	0.000
CELLULAR PROCESSES AND SIGNALING	Cytoskeleton	0.007	0.003	0.015	0.004	0.003	-0.008

INFORMATION STORAGE AND PROCESSING	RNA processing and modification	0.012	0.006	0.024	0.010	0.038	-0.012
INFORMATION STORAGE AND PROCESSING	Chromatin structure and dynamics	0.009	0.007	0.022	0.010	0.040	-0.013
METABOLISM	Carbohydrate transport and metabolism	8.600	0.355	8.705	0.765	0.814	-0.105
POORLY CHARACTERIZED	General function prediction only	11.801	0.194	11.932	0.350	0.461	-0.131
METABOLISM	Lipid transport and metabolism	2.331	0.089	2.473	0.157	0.084	-0.142
METABOLISM	Secondary metabolites	0.934	0.101	1.145	0.152	0.025	-0.210

	biosynthesis, transport and catabolism						
CELLULAR PROCESSES AND SIGNALING	Intracellular trafficking, secretion, and vesicular transport	2.072	0.155	2.328	0.273	0.079	-0.256
METABOLISM	Inorganic ion transport and metabolism	4.439	0.147	4.712	0.250	0.046	-0.274
INFORMATION STORAGE AND PROCESSING	Transcription	7.697	0.171	7.980	0.335	0.097	-0.283
CELLULAR PROCESSES AND SIGNALING	Cell motility	0.866	0.178	1.312	0.094	0.001	-0.446

CELLULAR PROCESSES AND SIGNALING	Signal transduction mechanisms	4.952	0.241	5.581	0.113	0.001	-0.628
POORLY CHARACTERIZED	Function unknown	6.795	0.388	7.506	0.614	0.043	-0.711
